# Tracking Fluorescent Dye Dispersion from an Unmanned Aerial Vehicle

**DOI:** 10.3390/s21113905

**Published:** 2021-06-05

**Authors:** Pawel Burdziakowski, Piotr Zima, Pawel Wielgat, Dominika Kalinowska

**Affiliations:** Faculty of Civil and Environmental Engineering, Gdansk University of Technology, 80-233 Gdańsk, Poland; piotr.zima@pg.edu.pl (P.Z.); pawel.wielgat@pg.edu.pl (P.W.); dominika.kalinowska@pg.edu.pl (D.K.)

**Keywords:** UAV, camera, fluorimeters, dispersion, fluorescein, rhodamine

## Abstract

Commercial unmanned aerial vehicles continue to gain popularity and their use for collecting image data and recording new phenomena is becoming more frequent. This study presents an effective method for measuring the concentration of fluorescent dyes (fluorescein and Rhodamine WT) for the purpose of providing a mathematical dispersion model. Image data obtained using a typical visible-light camera was used to measure the concentration of the dye floating on water. The reference measurement was taken using a laboratory fluorometer. The article presents the details of three extensive measurement sessions and presents elements of a newly developed method for measuring fluorescent tracer concentrations. The said method provides tracer concentration maps presented on the example of an orthophoto within a 2 × 2 m discrete grid.

## 1. Introduction

Unmanned platforms (water, land, and aerial) are devices able to move within a specific environment, without the need for an operator on-board. Their trajectory can be remotely controlled by humans or programmed and executed automatically [1,2,3]. Their essential advantage is the ability to execute a task in such regions, where a manned mission is difficult or impossible [2,4,5]. In the case of coastal management, unmanned platforms offer a very high spatial resolution [6,7,8] and flexibility [9,10]. These properties were quickly noticed by researchers and engineers, who started to use unmanned vehicles as mobile platforms for research equipment [11]. As a result, surveys have begun to be performed in new locations and with unprecedented frequency.

According to sources [12,13], approximately 80% of pollution in the marine environment is land-based. The contaminations reaching seas and oceans originate mainly from human activities conducted inland and, to a lesser extent, along the coasts [14]. The natural source and means of transport for all harmful substances and garbage from the hinterland to the sea are rivers [15,16]. Other sources include fishing, sea transport, and maritime infrastructure. Therefore, since rivers supply seas with pollutants, it becomes necessary to model the phenomena ongoing within the estuary zone and the propagation processes involving pollutants falling into salty waters [17].

Three methods are applied for studying the dispersion of pollutants within an environment, namely, tracer tests [18,19,20,21,22,23,24], determination of actual pollution [25,26], and mathematical modelling [27,28,29,30]. Tracer tests involve administering a safe substance (fluorescent dye) into the environment. The dyes mimic contamination, which enables obtaining information at the time of arrival, peak concentration, and the dimensions of the dissolved component cloud floating in a creek or receiver. Fluorescent dye concentration distribution is analyzed at the adopted points, which is a method of determining medium dispersion. Mathematical modelling is based on determining dispersion parameters [31], however, it requires feeding the model with data, its later verification, and fine-tuning. These models also enable predicting the dispersion of a given pollutant, which in turn requires providing information on the substance dispersion velocity and intensity field within a given environment. It is essential especially for some regions, where the environment is dominated by some specific physical process and the mathematical methods produce inaccurate solutions [29]. The mathematical modelling of the dispersion of pollutants cannot be separated from the spatial context of the natural environment, therefore an integrated modelling approach is used to understand the main environmental mechanisms governing the environmental contamination [17]. The integrated approach combines numerical models and spatial environmental data (i.e., bathymetry, digital terrain models).

A zone where fresh water mixes with salt water experiences water density changes, as well as profile velocity changes from gradient to drift [32,33,34]. The dynamics of this process is impacted by numerous factors, with the most important including wind, current, water density difference, salinity, and geomorphological river mouth conditions. Under such conditions, the dispersion process is very variable and can follow different steps. A precise determination of these parameters, hence, studying the pollutant dispersion, becomes a complicated task. Without a properly determined dispersion coefficient, mathematical models provide inaccurate results [29,35]. Determining the parameters describing the dispersion process is a prerequisite for obtaining the correct results of mathematical modelling [31] and procurement of a fully functional model [27,28,29,36]. One of the methods is tracer study with the use of passive and non-degradable substances as tracers.

Tracer tests are applied in various fields of science. They gained the greatest popularity in medicine and biotechnology [37,38,39,40], which utilize various substances, even the ones containing radioactive isotopes [41,42]. Tracers are used for studying protective clothing tightness [43] and analyzing nutrient migration in soils [43,44]. They can also be involved in testing water flow within an environment, or, in other words, widely understood as hydrology [45]. In this case, it is important for the marker not to have an adverse effect on the natural environment and that its detection is relatively simple. Fluorometry is a very popular method of tracer testing. It is based on fluorescent dyes as marking substances. Its main advantage is the easy detection of markers using fluorometric methods, simple determination of concentration over a wide range of values, and the fact that it is environmentally neutral. These studies involve two main substances, namely, Rhodamine WT (water tracer) and uranine, although there may be more [45]. The physical and chemical properties of these substances make them passive, nonreactive, and non-toxic in concentrations used for tracer testing [46]. Fluorescent dye molecules absorb electromagnetic radiation in the visible light range with a specific wavelength and emit radiation of lower frequency, in accordance with the Stokes law. The intensity of the emitted radiation is proportional to the concentration. Every dye is characterized by a specific excitation and emission spectrum. The outcome of tracer measurements is information on the change of concentration over time, at a selected point. Moreover, detecting and marking fluorescence is relatively simple, therefore, they have been used in hydrology since the 1960s.

Fluorometry is very popular in measuring the dispersion of various substances within the natural environment. Classically, the tracer concentration is spot-tested by sampling the carrier substances at a specified location. This method is useful, however, in the case of spatially extensive measurements, simultaneous sampling at many points is very challenging, and even impossible at times. As mentioned before, tracer dispersion in medicine and biotechnology is successfully diagnosed using image-based methods. As a result, it is not only possible to determine the measuring point concentration, but also to determine the front and its shape [47] and specify the direction and speed of the dispersion of these components. Therefore, it can be concluded that image-based methods are able to detect the concentration and location of fluorescent markers, and hence can be used for local tracer dispersion testing in surface waters using data obtained from unmanned aerial vehicles.

The still-niche approach to detecting fluorescent tracers from UAVs has been presented in several researches. The authors of [48] described a method for detecting Rhodamine WT based on a hyperspectral imaging system with 270 spectral channels mounted on UAV. Clark et al. in [49], similarly to the research presented by Legleiter et al. in [48] describes an airborne detection method using a multispectral camera fixed on an aircraft. Some researchers combine the technologies of unmanned aerial and surface vehicles to conduct tracer tests. Powers et al. [50] presented a combined technique of uranine detection and testing using a visible light camera, whereas a fluorometer was located on an unmanned surface vehicle (USV). What characterizes these tests from the previous ones is the camera type. A visible light camera, commonly referred to as a RGB camera, does not have precisely defined spectral channel values. Three quite wide channels are recorded in the red (R), green (G), and blue (B) visible part of the electromagnetic spectrum. Moreover, precise spectral characteristics are not available for such cameras, in the case of commercial, small UAVs. Not only multispectral and visible light cameras are used for fluorescent detection. Interesting research is the subject matter of [51], which involved using LIDAR (light detection and ranging) for detecting the natural fluorescence of marine corals. Baek et al. in [52] presents a tracer detection method (Rhodamine WT) based on RGB images. As indicated, RGB images exhibited a strong correlation with Rhodamine concentration. Furthermore, neural networks were used to convert the values recorded on images to concentrations.

This work describes a new approach for detecting and calculating the concentration of fluorescent tracers from a commercial UAV using a simple digital camera. The described method independently detects two dyes, namely, Rhodamine WT and uranine. The method were tested in real conditions, based on two different data collection approaches.

The publication is divided into four sections. The first section is the Introduction, which presents the basic motivation behind the research. The second section, Materials and Methods, describes the tools and methods used to process the data. Section three presents and discusses the obtained results. The article ends with the conclusions, where the most important aspects of the research are discussed and summarized. 

## 2. Materials and Methods

In the following section, the image-based method for fluorescent dye concentration measurement is described. The method was tested in three extensive measurement sessions where two different image data acquisition plans were performed and evaluated. During the experiments, two different fluorescent dyes were used. The research plan describes the data processing sequence. The sections end with the measurement object description and methodology used for in-situ measurement and procedure used for UAV flight.

### 2.1. Method for Image-Based Measurement of Dye Concentration

The method presented in this section has been developed for the purpose of measuring fluorescent dye concentration based on an image obtained from a RGB camera with a CMOS sensor, installed onboard a commercial UAV. Based on the CMOS sensor, it is already achievable to construct a relatively accurate fluorescence measurement instrument [53]. However, in this case we do not have active reagent excitation available. Based on simple methods and inspired by the solution proposed in [50], the authors developed a filtration algorithm and implemented it within a geographic spatial information system, while obtaining a geographic position measurement.

The image (*I*) recorded by a camera sensor is a discrete element matrix i×j for λ spectral channels. A CMOS camera installed onboard a Mavic Pro captures the image using three channels (R, G, and B) of unknown spectral characteristics, which can be expressed as:

I(i,j,λ)=r(i,j)+g(i,j)+b(i,j),

Based on average spectral characteristics for cameras [53,54,55,56] and a typical emission characteristic for the used dyes, it can be concluded that the main channels where the strongest spectral response is to be expected are g(i,j) for fluorescein (fluorometer-recorded wavelength at 540 nm) and r(i,j) for Rhodamine WT (fluorometer-recorded wavelength at 610 nm). The full sunlight spectrum, which naturally reaches the earth’s surface, is responsible for dye excitation in open waters and under daytime conditions. Therefore, by creating a difference in the spectral responses between the primary channels and others, the image of relevant dye can be enhanced. The differences for fluorescein can be expressed:

(1) $$d_{gb}\left(i,j\right)=g\left(i,j\right)-b\left(i,j\right),$$

(2) $$d_{gr}\left(i,j\right)=g\left(i,j\right)-r\left(i,j\right),$$

where $d_{gb}\left(i,j\right)$ represents the difference between the green (G) and blue (B) channels, $d_{gr}\left(i,j\right)$ represents the difference between the green (G) and red (R) channels. The differences for Rhodamine WT can be written:

(3) $$d_{rb}\left(i,j\right)=r\left(i,j\right)-b\left(i,j\right),$$

(4) $$d_{rg}\left(i,j\right)=r\left(i,j\right)-g\left(i,j\right),$$

where $d_{rb}\left(i,j\right)$ represents the difference between the red (R) and blue (B) channels, $d_{rg}\left(i,j\right)$ represents the difference between the red (R) and green (G) channels.

To avoid negative values for the sum of spectral channel differences, the sum of spectral channel differences, which additionally emphasizes the image of the dye itself, can be expressed (respectively, for fluorescein and rhodamine) as:

(5) $$f\left(i,j\right)=\sqrt{{\left(d_{gb}\left(i,j\right)+d_{gr}\left(i,j\right)\right)}^{2}},$$

(6) $$r\left(i,j\right)=\sqrt{{\left(d_{rb}\left(i,j\right)+d_{rg}\left(i,j\right)\right)}^{2}}.$$

Assuming the existence of a background fluorescence peak value, namely, non-zero values for areas with no dye on the image, all results (4) and (5) below the threshold *t* shall be assigned with 0, which can be respectively written as:

(7) $$f_{nr}\left(i,j\right)=\left\{f\left(i,j\right)<t\Rightarrow 0,f\left(i,j\right)\geq t\Rightarrow f\left(i,j\right)\right\},$$

(8) $$r_{nr}\left(i,j\right)=\left\{r\left(i,j\right)<t\Rightarrow 0,r\left(i,j\right)\geq t\Rightarrow r\left(i,j\right)\right\}.$$

The last calculation stage was result filtering, which slightly averages the values:

(9) $$f_{f}\left(i,j\right)=f_{nr}\left(i,j\right)*c\left(m,n\right),$$

(10) $$r_{f}\left(i,j\right)=r_{nr}\left(i,j\right)*c\left(m,n\right),$$

where * is a discrete convolution and c(m,m) is a Gauss-masked kernel, which can be written:

(11) $$c\left(m,n\right)=e^{-\frac{\left(m^{2}+n^{2}\right)}{2\sigma^{2}}},$$

where σ is the standard deviation and is a variable that defines the filtration strength.

The values of matrices $f_{f}\left(i,j\right)$ and $r_{f}\left(i,j\right)$ contain a digital number corresponding to the intensity of radiance. Therefore, the last stage of the calculations will be converting from a radiance value to the concentration of a given dye. For this purpose, the authors compared the values of matrices $f_{f}\left(i,j\right)$ and $r_{f}\left(i,j\right)$ at specific positions, where the values were measured with a fluorometer. This created a matrix containing the values of $f_{f}\left(i,j\right)$ and $r_{f}\left(i,j\right)$, such that (i,j)=(x,y), where (x,y) is the position of in-situ measurements, which is the sampling position. The Vandermond matrix was used to calculate the values of $p_{1}$, $p_{2}$ and $p_{3}$, and the calibration polynomial [57]. Therefore, the radiation value expressed by dye concentration can be written as follows:

(12) $$f_{c}\left(i,j\right)=p_{1}^{2}f_{f}\left(i,j\right)+p_{2}f_{f}\left(i,j\right)+p_{3},$$

(13) $$r_{c}\left(i,j\right)=p_{1}^{2}r_{f}\left(i,j\right)+p_{2}r_{f}\left(i,j\right)+p_{3}.$$

### 2.2. Research Plan

**Figure 1:**
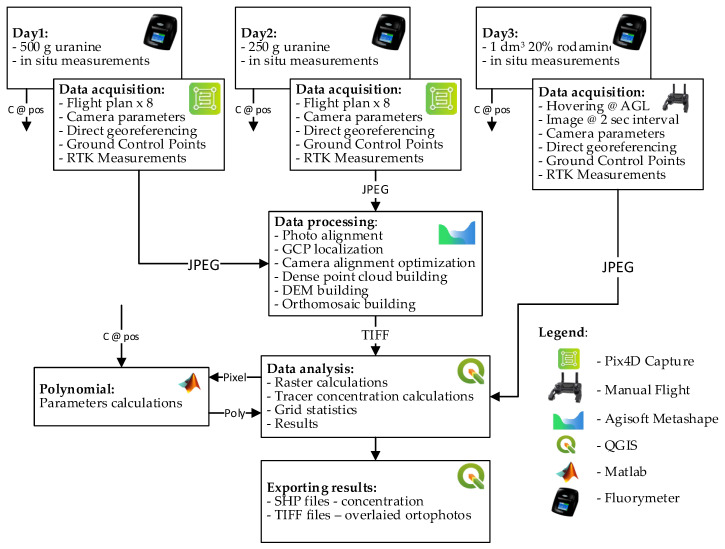
Research and result elaboration flowchart (C @ Pos—Concentration at position).

In order to verify the procedure presented above and the research assumptions of this article, a test schedule and a computation process were developed. This process is in graphical form, and the used tools are shown in Figure 1. Test data was acquired using a commercial UAV with a camera and a fluorometer. The study period involved completing three full test sessions in several week intervals. Each session comprised of several UAV flights at regular time intervals, and simultaneous manual sampling for fluorometer testing at strictly specified spots. The research data was processed using popular photogrammetric software, followed by geoinformation software. Simultaneously, all collected water samples were independently assessed in terms of dye concentration. The UAV flights within the research were planned using two different methods, to evaluate the flight plans for these measurements at a later time. The details of these operations are shown in the further section of this research.

### 2.3. Measured Object

**Figure 2:**
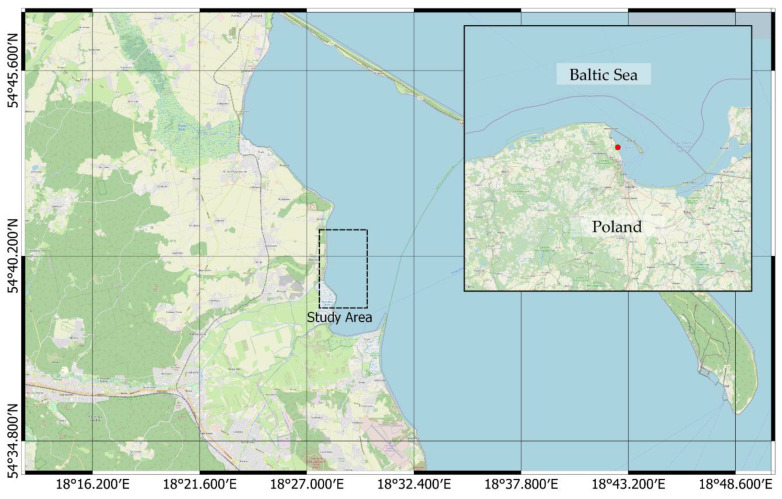
Study area, catchment of the Gizdepka river, Bay of Puck, Southern Baltic Sea (coordinate reference system: WGS84).

A minor watercourse, with a catchment area of 37.2 km^2^, called Gizdepka, was selected for the study [58,59]. The average annual flow rate falls within a range of 0.16 to 0.19 m^3^/s. This watercourse flows directly to the Bay of Puck (Southern Baltic Sea) (Figure 2), hence supplying the bay with pollutants and biogenic compounds. Gizdepka passes through an agricultural area, which results in periodic increases of biogenic compound supply, including significant concentrations of nitrogen and phosphorous compounds [60].

### 2.4. Fluorometer Measurement

**Figure 3:**
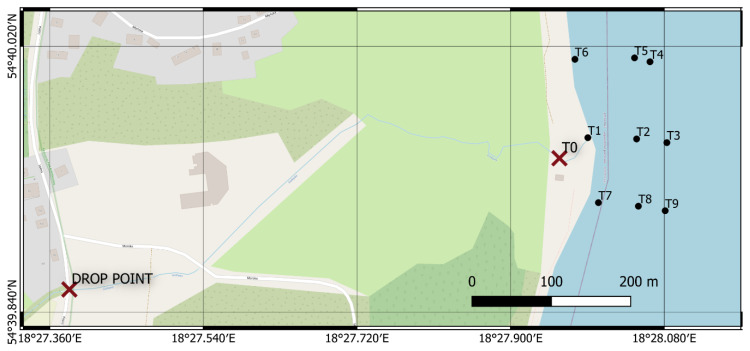
Research area, and the positioning of GCPs, reference poles and the drop point (coordinate reference system: WGS84).

The tests were scheduled and conducted in the early Spring of 2019, when the chlorophyll concentration in the water is the lowest and water transparency is as high as possible. Three full measuring sessions were conducted at two-week intervals. Experimental condition data, as well as dye concentrations and type are shown in Table 1. In each case, the dye was released into the river as an impulse at a drop point, located 750 m from the river mouth (Figure 3).

The study involved using uranine (fluorescein sodium) and Rhodamine WT (water tracer) marker substances. These are xanthene fluorescent dyes. The dyes absorb light of the relevant wavelength and emit light of a longer wavelength. This means that the fluorescence band will be in the lower frequency range than the absorption band and will be moved towards the red band. The maximum excitation and emission wavelengths for Rhodamine WT are 558 and 582 nm, respectively, and 419 and 512 nm for uranine. Fluorescence intensity of a specific solution is proportional to its concentration. Furthermore, the selected dyes are characterized by good solubility, low reactivity, poor absorption in a suspension, unmodifiable medium properties and are nontoxic in concentrations used in the research.

After releasing the dye to water, the scientists commenced sampling the water at specific points and times. Water samples were collected at the river mouth, with a varying time step, selected to capture concentration changes at the time of peak concentration, in particular. Samples in the coastal zone were collected at spots that were physically marked with flagpoles. Marking the spots with poles allowed to precisely indicate the sampling position and their later identification on the measurement images. After a session was completed, the samples were transported to a laboratory, where the dye concentration in each of the samples was thoroughly evaluated. Dye concentrations were determined using the “Trilogy” fluorometer (Turner Designs, San Jose, CA, USA) (Table 2). This provided information on the change in the concentration of the migrating dye over time in the river mouth (position T0) and selected points in the bay around the Gizdepka estuary (positions T1 to T9) (Figure 3).

**Figure 4:**
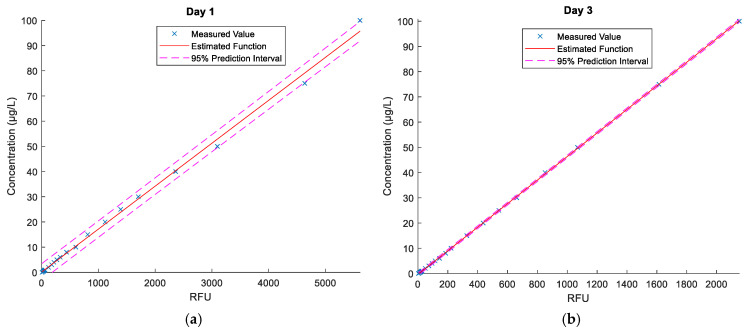
Estimated calibration curves for measurements with the “Trilogy” fluorometer, (**a**) fluorescein (**b**) Rhodamine WT.

The fluorometer measures fluorescence in relative fluorescence units (RTUs). Calibration curves for Rhodamine WT and uranine, based on the procedures of the United States Geological Survey [61]. Each calibration curve enables converting relative fluorescence units to concentration ($C_{f}$) expressed in μg/dm^3^, using a linear function $c_{f}=f\left(RTU\right)$ for a range of 0 to 100 μg/dm^3^. Furthermore, prior to injecting the dyes, reference zero samples from the bay and the river were collected to determine fluorescence background. Based on known fluorescence levels, the water fluorescence in the samples collected in the course of the measurements was determined and the results were converted using a calibration curve. Environmental samples with a concentration beyond 100 μg/dm^3^ were diluted to a range of a corresponding calibration curve in each case. Estimated calibration curves for the fluorometer are shown in Figure 4. The curves were determined in a laboratory, based on the reference samples.

### 2.5. UAV Measurement

Photogrammetric flights were conducted using a DJI Mavic Pro (Shenzhen DJI Sciences and Technologies Ltd., China) UAV. This unmanned aerial vehicle is representative of commercial aerial vehicles, designed and intended primarily for recreational flying and for amateur movie makers. The versatility and reliability of these devices were quickly appreciated by the photogrammetric community. Their popularity results mainly from their operational simplicity and very intuitive ground station software. The UAV is equipped with an integrated digital camera with a CMOS (Complementary Metal-Oxide-Semiconductor) sensor. The FC220 digital camera installed onboard this UAV is a compact device with a very small 1/2.3” (6.2 × 4.6 mm) sensor and a minor maximum ISO (1600) sensitivity. Images are recorded in the visible light band and using the JPEG or DNG format. They are typical images, recorded in three channels, namely, RGB (red, green, blue). Since these types of cameras are mostly used for filming and photographic activities, full specifications are not available, as in the case of multispectral survey cameras. The manufacturer does not provide precise spectral characteristics [62], and the individual spectral channels record the bands over a very broad range. Furthermore, there is also no official information on the type of used sensors (manufacturer name, type, catalogue number), besides only the indicated execution technology, namely, CMOS. It should be presumed that it has a specification typical for popular CMOS sensors, however, with a clearly filtered near-infrared band above 780 nm. The application of a near-infrared filter is a standard manufacturing practice for such cameras; therefore, this should also be assumed in this case. The methodology presented here does not depend on accurate spectral characteristics and enables the utilization of commercially available RGB cameras for this type of survey.

Several UAV flights and sessions were conducted on each day of the measurements. The flights were conducted at regular time intervals, as the coverage area contains the tracer trail visible on water. On Days 1 and 2, the UAV conducted flights following a programmed single-grid trajectory, as in the case of the developed orthomosaic. On Day 3, the measurement was taken using a different technique—the drone hovered in the air and took an image at a constant interval (up to 2 s). Simultaneously, the operator matched the AGL altitude and hovering positions of the UAV, so that the area of a single image covered the entire area of the visible dye. In each case, the camera was always positioned in the nadir, with no oblique images taken.

The measurements involved developing a photogrammetric network that comprised of eight ground control points (GCPs), arranged along the river, and their position was measured with the GNSS RTK accurate satellite positioning method. The GCP position was determined relative to the PL-2000 Polish state grid coordinate system and their altitude relative to the PL-EVRF2007-NH quasigeoid. All checkpoints were marked using special boards. The position of the poles placed within the water section of the research area was also measured using the GNSS RTK method.

**Figure 5:**
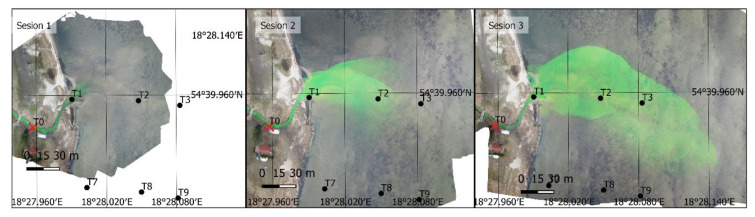
An orthophoto depicting the first three sessions on Day 1 (fluorescein).

**Figure 6:**
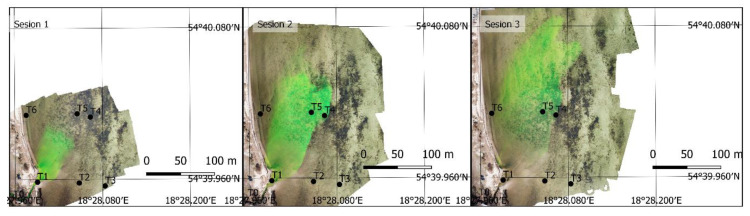
An orthophoto depicting the first three sessions on Day 2 (fluorescein).

**Figure 7:**
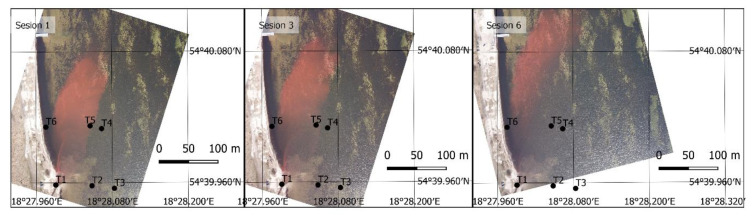
An orthophoto depicting sessions 1, 3, and 6 on Day 3 (Rhodamine WT).

Image data collected on Days 1 and 2 were used to develop an orthomosaic. The result included 16 orthomosaics, mapping the dye position on Days 1 and 2. The orthophoto taken on Days 1 and 2 for the first three sessions are shown in Figure 5 and Figure 6. On Day 3, A simpler, single-image photogrammetry method was used in this case. Each image was rectified and fitted using GCPs located in the images and measured in the field. This resulted in obtaining a satisfactory imaging accuracy, while simultaneously enabling recording tracer dispersion at any interval. As a result, 369 images (every 2 s) were recorded and 8 images depicting tracer dispersion were rectified on Day 3. Only the images taken at the same time as the sampling time were rectified. The results for sessions 1, 3, and 6 are shown in Figure 7.

## 3. Results

In the Results section, the main findings of the article are summarized. The relevant graph presents concentration in the time domain, while the UAV measurements were presented in the cartographic form. The in-situ measurements, performed using the laboratory fluorometer, were compared with the results obtained from the RGB images.

### 3.1. Fluorometer

**Figure 8:**
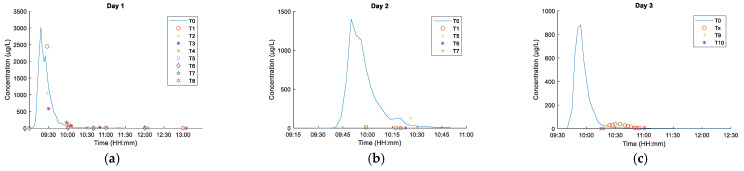
Dye concentration at checkpoints, measured with a fluorometer. (**a**) Day 1, fluorescein, (**b**) Day 2, fluorescein, (**c**) Day 3, Rhodamine WT.

Figure 8 shows the fluorometer measurement results for individual measurement sessions covering the river mouth (T0) and positions T1–T9.

As seen from the graphs, collecting numerous samples from the Gizdepka river mouth (T0) enabled accurately determining the concentration shift curve and the time of the peak. Spot measurements conducted within the measurement field marked with poles (T1 to T9) allowed to determine the local concentration at a given time.

Traditional sampling and measurement enabled collecting information on local concentration changes within a one-dimensional object, such as a river. The dye was released to the watercourse as an impulse, at a distance of 750 m from the reference section located at the river mouth (station T0). As a result of longitudinal dispersion, the impulse was transformed into a long dye trail. It took approximately 1 h for the trail to pass the reference section. The shape of the obtained concentration curves at the reference section (Fg) is associated with the hydraulic characteristics of the watercourse, among others, heterogeneous creek speed distribution and the natural nonuniform and transient flow occurring in natural watercourses. Traditional measurements proved to be good for capturing concentration changes at the river mouth (station T0).

On the other hand, the dye dispersion in the bay was very dynamic. The changes were of several orders of magnitude over a relatively short distance to the nearest position (T1–T9). This results in much lower concentrations within the measurement field. Capturing the spatial distribution using the traditional method is difficult, since it requires measuring at many points simultaneously. The measurement turned out to be difficult to conduct, even in the conditions of a shallow bay. The use of submersible fluorimetry sensors may be impossible due to the susceptibility of wired data transmission to wave-induced damage. This creates a potential advantage for remote sensing methods in recording the phenomenon.

### 3.2. UAV Measurements

**Figure 9:**
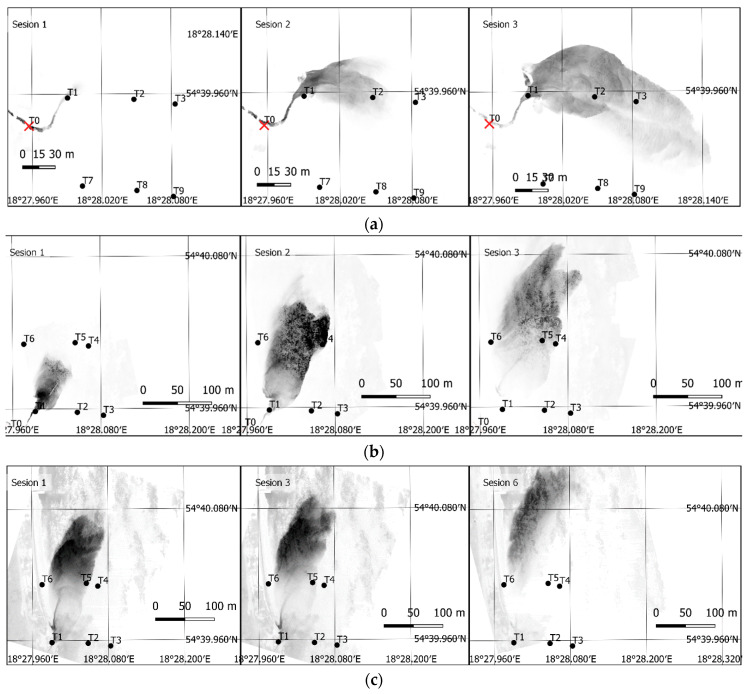
Post-filtration images, without converting pixel values to dye concentrations. (**a**) Day 1, sessions 1–3 (fluorescein), (**b**) Day 2, sessions 1–3 (fluorescein), (**c**) Day 3, sessions 1, 3, and 6 (Rhodamine WT).

According to a previously described procedure and based on image $f_{f}$ and $r_{f}$, results of Equations (8) and (9), respectively, pixel values in position (i,j) were collected, in accordance with the field sampling position (x,y). These images, resulting from Equations (8) and (9) for similar sessions presented in Figure 5 and Figure 7 are shown in Figure 9.

As seen in these images, the presented filtration method enables correctly extracting the dye position. The applied difference in spectral channel radiance values allows to minimize the impact of other channels on the image, thus emphasizing the fundamental channel for a given dye. They are the green (G) channel for fluorescein and red (R) for rhodamine. The presented image shows that tracer dispersion in this area forms a clear border with a stepwise change of dye concentration in the side sections of the moving cloud. The method used in this case shows this borderline very well. Dye cloud front concentration changes slowly and is not as stepwise anymore. The remote sensing method using small UAV is characterized by a rather low sensitivity of low concentration detection, hence the dye wave front depicted on the air images can be slightly shifted backwards, opposite to the main dispersion direction.

In the case of fluorescein, the extraction is clearer, the bottom sediments are not as intensely visible as they are for case (b) in Figure 9. It should be added that the water depth in the study area is low, up to 1.5 m. On a sunny day, in late winter or early spring conditions, the water clarity is high enough to see the bottom very easily, including the deposited sediments. The bottom image creates a distinct background for the dye and also creates some low levels of radiance in that region, subsequently being converted to dye concentration.

**Figure 10:**
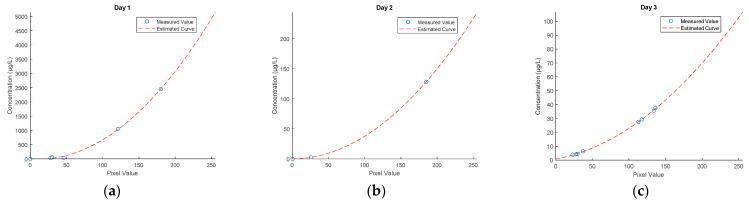
Calibration curves for an RGB camera. (**a**) Day 1, fluorescein, (**b**) Day 2, fluorescein, (**c**) Day 3, Rhodamine WT.

Figure 10 shows the graphs of calibration polynomials calculated as per Equations (11) and (12), which allowed converting the radiance values to dye concentration values. As mentioned in [61], dye dispersion air testing is a very good method, however, the dye must be visible to the human eye, and hence, to the digital camera sensor. Unlike a fluorometer, which is a very sensitive device and records residual fluorescence, the minimum concentration values are raised to an amount of approximately 10 to 20 µg/dm^3^. In the case of lower concentration values, the used camera and filtration method do not enable precise extraction of the dye cloud. As noted during the tests, the fluorometer already recorded an increase in dye concentration, especially at the cloud front, however, such low concentrations were not visible in the images.

### 3.3. Final Results

**Figure 11:**
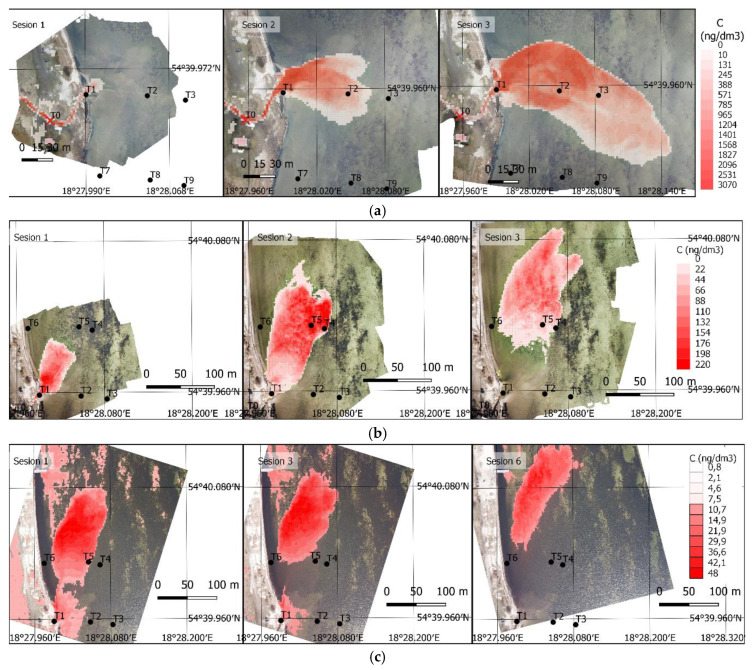
Final results, a grid with the mean dye concentration. (**a**) Day 1, sessions 1–3 (fluorescein), (**b**) Day 2, sessions 1–3 (fluorescein), (**c**) Day 3, sessions 1, 3, and 6 (Rhodamine WT).

The ground sampling distance (GSD) of the presented photogrammetric images is approximately 3 cm. Therefore, it is possible to calculate the dye concentration for minimal grid size equal GSD. Most numerical models do not require such high data resolution. Therefore, a regular grid, 2 × 2 m was established, and a mean concentration value was calculated for its single cells. This additionally contributed to minimizing the impact of noise and outliers on the concentration value for a given grid cell. The final results, in the form of a regular 2 × 2 m grid showing a mean dye concentration value for a given cell, are listed in Figure 11. The concentration grid was projected on a layer of the orthophoto for the case on Days 1 and 2, and on the rectified images for the case on Day 3. This allows to clearly see the geographic context and change dynamics for a given dispersion case.

The aforementioned method enables obtaining tracer distribution in space and over time. The verification calculations involving the transport of a passive dissolved substance can follow several methods. One of them is to conduct statistical calculations. The second method is to run numerical calculations aimed at solving an advection dispersion equation. An image of a 2 × 2 m grid taken at the moment of releasing the tracer in the river mouth can be used as a starting condition, and the calculations can be conducted over time. In such a case, the remaining images will constitute material for comparison with the obtained results. The calculations can also be conducted assuming each obtained image as the starting condition and the next image as the comparative material. This leads to obtaining several, usually slightly different, transport parameter sets (including dispersion coefficients), and to determine the mean values. It seems that the second approach is more rational. The first approach usually assumes a large starting concentration, followed by running the calculations. Unfortunately, in the case of high concentrations, reading this from the image might differ relative to the reality. Subsequent sequences can exhibit far-reaching differences. This flaw can be corrected in the second approach. Assuming an incorrect concentration at the beginning is corrected during subsequent sequences, and the mean dispersion parameters are the outcome.

Finally, Table 3 and Table 4 present control values for fixed stations at certain fixed stations. The dye concentration measured at fixed station (C $(\mu g/dm^{3}$)) is compared to the calculated values form the presented method. Value $f_{c}\left(i,j\right)$ represents calculated concentration at certain pixel position with the specific value, resulting from Equations (8) and (9). The mean $f_{c}\left(i,j\right)$ represents a mean dye concentration value for a given cell (2 × 2 m).

## 4. Conclusions

The article presents an effective method for measuring the concentration and dispersion of fluorescent dyes using a UAV. This method is based solely on an image obtained from a commercial RGB camera and a calibration measurement taken with the use of a fluorometer. It enables to quickly estimate the concentration values for the entire surface of the tested dye, in any spatial resolution, not lower than GSD.

Two different approaches were tested within the study. The first on Days 1 and 2 and the second on Day 3. Typical photogrammetric flights following a single grid were used on Days 1 and 2, thus constructing an orthomosaic. Such a flight plan enables mapping a relatively large area from a low altitude. This, in the context of legal restrictions regarding UAV flight altitude enables covering the required area, however, it takes a little longer. In situations where the dispersion is very quick and rather dynamic, such a flight plan can result in incorrect mapping of the dye cloud shape. Furthermore, in the case of basins without visible bottom, it will be impossible to find tie points on the images and develop an orthophoto. Similar to the case of [7], a water surface orthophoto will not be possible to develop. On the other hand, the bottom image, especially with visible sediments, forms a clear background for the dye and, in the case of low concentrations, will hinder the filtration and extraction of the dye cloud. The solution applied for Day 3, namely, UAV hovering over the area and taking images at a set interval, allows to track the dynamics very accurately, with a preset time resolution. The time resolution for Day 3 was 2 s, while it amounted to 15–20 min for Days 1 and 2. In this case, the camera was positioned in the nadir, therefore, the area coverage of the image had to be adjusted by the flight altitude. The flight area for large areas of monitored dispersion can be significant, therefore, this might constitute a certain restriction. In this case, the flight altitude for Day 3 tests changed from 150 to 350 m AGL (above ground level). Of course, one can use a wide-angle lens, thus reducing the flight altitude.

In the first approach, the relatively long time to acquire images for an orthomosaic building has become a challenge. The flight takes from a few to a dozen of minutes, depending on the size of the tracer cloud. This can cause some difficulties in the synchronization of in-situ measurements, and with a very dynamic changing, even make precise measurements impossible. In the second approach, where the UAV remains hovering over the study area and simultaneously captures the entire tracer cloud, covering its entire area in the image, a low interval can be set. This allows to record very dynamic changes, while synchronizing more accurately with the in-situ measurements.

In the case of lower concentration values, the used camera and filtration method do not enable precise extraction of the dye cloud. Ten µg/dm^3^ was adopted as the limit minimum concentration value. Below this value, the dye becomes invisible to the camera and thus, it was impossible to extract the dye image. These results confirm the thesis set in [61], where the authors clearly indicated that tracking a tracer from air is possible in the case of concentrations that are directly visible to the human eye, hence, to the camera lens. For higher concentrations, which already impossible to directly measure with a fluorometer, this method provides good results. Dye concentrations above 50 µg/dm^3^ are already clearly visible, and extreme concentration even above 1000 µg/dm^3^ has no significant impact on detection.

The measurement on a cloudy day (Day 1) turned out to be the most effective. The bottom image in clear water was minimal, which enabled precisely extracting the dye cloud shape. Water transparency turns out to be a factor hindering UAV measurements and subsequent data extraction following the described method. Furthermore, a measurement on a sunny day poses a risk of strong water surface reflections, which in extreme cases may make taking an image impossible.

The described method does not require calibration based on typical tables for radiometric calibration. Direct dye concentration measurement with a fluorometer was used for calibration. This method requires additional field measurement and does not enable to objectively determine the concentration based only on the image, as in the case of typical remote sensing. The difficulty in this case is the lack of spectral characteristics of the used camera, which is why this approach provides results, while requiring the use of a fluorometer. Furthermore, a similar approach was also presented in [50], with the difference being the use of a USV for sampling with a fluorometer.

This data can be used to calibrate or verify dispersion models. The method for processing an image of concentration enables obtaining spatial, time-variable concentration distributions on a discrete grid. Dispersion parameters can be obtained by applying stochastic methods or conducting numerical calculations. Both methods enabled obtaining good results.

## Figures and Tables

**Table 1:** List of measuring sessions using a fluorescent tracer.

Name	Date	Tracer	Tracer Concentration
Day 1	17 February 2019	Uranine	500 g/10 dm^3^ H_2_O
Day 2	2 March 2019	Uranine	250 g/10 dm^3^ H_2_O
Day 3	23 March 2019	Rhodamine WT	1 dm^3^ @ 20%

**Table 2:** Parameters of the Trilogy fluorometer.

Parameter	Rhodamine WT	Uranine
Minimum detected concentration	0.01 ppb	0.01 ppb
Linear range of concentration—fluorescence relationship	0–500 ppb	0–200 ppb
Excitation electromagnetic wavelength	550 nm	485 nm
Recorded electromagnetic wavelength	610 nm	540 nm

**Table 3:** Measured and calculated values for specific fixed stations (T1–T3)-Day 1.

**Sesion**	**Time**	**T1** $C\;(\mu g/{\mathrm{dm}}^{3})$	**Pixel** **Value**	$f_{c}\left(i,j\right)$	$\mathrm{Mean}\;f_{c}\left(i,j\right)$	$T2\;C\;(\mu g/{\mathrm{dm}}^{3})$	Pixel Value	$f_{c}\left(i,j\right)$	$\mathrm{Mean}\;f_{c}\left(i,j\right)$	$T3\;C\;(\mu g/{\mathrm{dm}}^{3})$	Pixel Value	$f_{c}\left(i,j\right)$	$\mathrm{Mean}\;f_{c}\left(i,j\right)$
1	09:43	715	103	718	7081	1	0	3	2,83	1	0	3	3
2	09:55	2449	180	2454	24,532	1052	121	1032	1042	1	0	3	3
3	10:07	2069	166	2062	2051	1331	135	1315	1322	584	93	568	567
4	10:28	55	30	21	201	40	46	95	84	285	69	278	270
5	10:58	12	25	7	3	12	0	3	3	70	42	72	62
6	11:26	10	0	3	3	8	0	3	3	26	28	15	21
7	12:02	7	0	3	3	7	0	3	3	1	0	3	3
8	13:03	6	0	3	3	5	0	3	3	1	0	3	3

**Table 4:** Measured and calculated values for specific fixed stations (T1–T3)-Day 3.

Sesion	Time	T1 $C\;(\mu g/{\mathrm{dm}}^{3})$	Pixel Value	$f_{c}\left(i,j\right)$	Mean $f_{c}\left(i,j\right)$
1	09:43	10:25	2,932,284	1176	30,002,168
2	09:55	10:28	2,758,726	1129	28,145,903
3	10:07	10:31	37,611,736	1362	37,911,632
4	10:28	10:34	35,541,976	1343	37,062,427
5	10:58	10:52	6,331,872	372	6,160,352
6	11:26	10:55	4,430,896	295	4,758,875
7	12:02	10:58	3,906,064	23	36,958
8	13:03	11:01	4,237,472	276	4,436,768
